# Right Ventricular Dilation in Cardiac Arrest May Have Complicated Implications: A Case Report

**DOI:** 10.7759/cureus.23608

**Published:** 2022-03-29

**Authors:** Di Coneybeare, Miles Gordon

**Affiliations:** 1 Department of Emergency Medicine, Columbia University Irving Medical Center, New York, USA

**Keywords:** cardiac physiology, prognosis, right ventricular dilation, cardiac arrest, point-of-care ultrasound

## Abstract

Right ventricular (RV) dilation has been observed in patients in cardiac arrest. Historically, this phenomenon is almost always attributed to massive pulmonary embolism. However, recent advancements have revealed that there are many other causes of RV dilation in cardiac arrest. In this case report, we present the case of an elderly woman who was found in cardiac arrest with an initial normal left ventricle to RV ratio with subsequent development of RV dilation in the midst of resuscitation without changes to other hemodynamic parameters. This case further bolsters the complex nature of cardiac physiology in cardiac arrest and the need for further investigation.

## Introduction

Recent studies have shown that right ventricular (RV) dilation in cardiac arrest can be caused by a myriad of factors that range from primary dysrhythmias to hyperkalemia, challenging the historical assumption that RV dilation in cardiac arrest equates to pulmonary emboli [1-4]. Further porcine models have demonstrated that RV dilation does not meaningfully differ regardless of the possible underlying cause, whether it be hypovolemia, hyperkalemia, primary dysrhythmias, or pulmonary embolism [3]. In the absence of these diagnoses, what is the significance of RV dilation on point-of-care ultrasound (POCUS) during resuscitation of the patient in cardiac arrest? And more importantly, what should be done when RV dilation is observed on POCUS in a patient in cardiac arrest? There are still many unknown physiological and anatomical changes that occur in a patient in cardiac arrest. Through further exploration and observation of structural changes in the cardiac arrest patient, we hope to improve our understanding of the underlying causes of structural cardiac changes and decrease future management ambiguity. Here, we describe a case in which a patient in cardiac arrest with an initially normal RV to left ventricular (LV) ratio develops RV dilation after the return of spontaneous circulation.

## Case presentation

A 79-year-old female with a past medical history of diabetes (DM), hypertension, and chronic kidney disease (CKD) presented status post-cardiac arrest with the return of spontaneous circulation (ROSC). On arrival, the patient had been intubated, and her initial vitals were blood pressure 128/77, pulse 106, respiratory rate 16, and SpO_2_ 96%. As per emergency medical services (EMS), the patient suffered an unwitnessed out-of-hospital cardiac arrest with no initial bystander cardiopulmonary resuscitation (CPR). EMS providers found the patient in a non-shockable rhythm by an automated external defibrillator (AED). After initiating CPR, EMS administered epinephrine and sodium bicarbonate. Ventricular fibrillation was subsequently noted on the AED; the patient was defibrillated and achieved ROSC. 

Shortly after arriving in the emergency department (ED), the patient again lost pulses, with ventricular fibrillation noted on the monitor. CPR was reinitiated and the patient was defibrillated. ROSC was confirmed by organized myocardial activity on POCUS, manual palpation, and vital sign measurements. The initial RV to LV ratio was observed as normal (Video 1), and a plethoric inferior vena cava (IVC) lacked respiratory variation (Video 2).

The patient again lost pulses several minutes after achieving ROSC with ventricular tachycardia followed by ventricular fibrillation on the monitor. The patient underwent CPR, multiple rounds of defibrillations in addition to receiving amiodarone, lidocaine, calcium chloride, and sodium bicarbonate with ROSC for the third time. During one of the pulse checks during the third resuscitation, the RV now appeared dilated (Video 3). After ROSC, the patient’s vital signs remained stable with oxygenation at 100% on the vent. Vent settings were on the volume control, respiratory rate set at 12, tidal volume of 450, positive end-expiratory pressure (PEEP) of 5, and FiO_2_ of 30%.

In consultation with cardiology, the decision was made to transfer the patient for catheterization to another hospital site. While awaiting transport on a norepinephrine drip, the patient decompensated, became hypotensive and subsequently pulseless for the fourth time. Unlike the prior resuscitation attempts, the rhythm progressed to and remained in asystole, and ultimately expired despite maximum resuscitative efforts.

**Video 1:** Initial normal right ventricular to left ventricular ratio.

**Video 2:** Dilated inferior vena cava.

**Video 3:** Right ventricular dilation.

## Discussion

The patient’s RV appeared larger than the LV in the third resuscitation and demonstrated severe RV dysfunction. RV dilation and dysfunction in cardiac arrest patients have historically been attributed to pulmonary emboli. In 2000, Comess et al. shined light on the fact that RV dilation occurs in cardiac arrest without the primary thromboembolic disease [5]. To our knowledge, this is the first case report capturing progressive RV dilation during the resuscitation of a patient in cardiac arrest with an initially normal RV to LV ratio while already in cardiac arrest for an extended period of time.

Many causative factors could contribute to RV dilation and dysfunction. Pulmonary embolism was low on the differential as the patient did not have escalating oxygen requirements and remained normoxic on the ventilator throughout the resuscitation. Studies have elucidated that hyperkalemia, hypovolemia, and hypoxia could also cause dilation [1-3]. Hyperkalemia was part of the differential, as our patient suffered from CKD, and therefore, we administered calcium chloride. Ultimately, laboratory findings did not reveal hyperkalemia. Hypovolemia was also lower on the differential as she received crystalloids by EMS as well as throughout many of her resuscitation, and had a plethoric IVC prior to RV dilation.

Prolonged dysrhythmia, if left untreated, has also been shown to dilate the RV in swine models and was considered as a part of the differential for RV dilation [4]. However, the mechanism in which dysrhythmia, specifically untreated ventricular fibrillation, contributes to RV dilation is thought to be due to stroke volume deficiency from the LV; the lack of adequate forward movement of blood causes equilibration of pressures on both sides of the heart, leading to RV dilation [4,6]. These findings have led some authors to stress the importance of chest compressions prior to defibrillation [4,6]. However, our patient was initially in a non-shockable rhythm and received chest compressions within seconds of detecting ventricular fibrillation making, untreated prolonged dysrhythmia less likely.

Another possible differential for the patient’s RV dilation could reside in the patient’s ultimate prognosis. The RV differs from the LV in its circulatory supply as well as metabolic demand, making it generally more resilient to ischemic insult compared with the LV [7]. Ramjee et al. found that severe RV dysfunction after ROSC, regardless of LV dysfunction, in patients who survived hospitalization represented an independent predictor of worse neurologic and mortality outcomes [7]. Therefore, the presence of severe RV dysfunction in the face of no other reasonable explanation may be a harbinger of poor morbidity and mortality. When the situation offers no clear underlying cause, prognostic aspects of RV dilation serve as an intriguing possibility, especially as it has been found that RV dilation exists to some degree in most cases of cardiac arrest regardless of the underlying cause [8].

## Conclusions

To our knowledge, this is the first case report to demonstrate dynamic RV dilation in a patient with cardiac arrest. As cardiac morphology during cardiac arrest is a relatively new field of research, human data on this topic remains limited. Extrapolating from established animal data, this case illustrates the possible broad differential diagnosis when faced with dynamic RV dilation in a patient with cardiac arrest. Cardiac arrest physiology remains a wide-open field of study, especially with regard to right ventricular function. We hypothesize that in the absence of an alternate etiology, progressive RV dilation during cardiac arrest may be a marker of poor prognosis. The proliferation of emerging technologies such as transesophageal echocardiography can provide further evidence in the clinical setting. Future studies can transition from porcine models to evaluation of cardiac morphology in patients with cardiac arrest in conjunction with clinical data.
